# Daptomycin-Associated Acute Eosinophilic Pneumonia: A Case of a Severe Reaction

**DOI:** 10.7759/cureus.83157

**Published:** 2025-04-28

**Authors:** Ibrahim Shamasneh, Neaam Al-Bahadili, Samuel K Dadzie, Nicholas Fox

**Affiliations:** 1 Internal Medicine, Piedmont Athens Regional Medical Center, Augusta University/University of Georgia (AU/UGA) Medical Partnership, Athens, USA; 2 Pulmonary and Critical Care Medicine, Piedmont Athens Regional Medical Center, Augusta University/University of Georgia (AU/UGA) Medical Partnership, Athens, USA

**Keywords:** acute eosinophilic pneumonia, bronchoscopy, daptomycin, daptomycin-induced acute eosinophilic pneumonia, glucocorticoids

## Abstract

Acute eosinophilic pneumonia (AEP) is a condition characterized by an excess of eosinophils in the interstitial and alveolar spaces, often linked to exposure to agents like inhalants, non-steroidal anti-inflammatory drugs, tobacco smoke, and, on rare occasions, daptomycin. It manifests with fever, dyspnea, hypoxia, and abnormal findings on computed tomography (CT) or radiography. Misdiagnosing AEP as community-acquired pneumonia or malignancy can delay treatment. Bronchoscopy provides a definitive diagnosis, typically with an elevated eosinophil count exceeding 25% on bronchoalveolar lavage (BAL). We are presenting an 80-year-old Caucasian male with osteomyelitis of the foot on daptomycin, who presented three weeks later with acute-onset dyspnea and a mild grade fever. A CT scan of the chest revealed bilateral ground-glass opacities. The closely associated timing and the absence of identifying an infectious etiology raised concern for daptomycin-induced AEP. Given our high clinical suspicion, bronchoscopy was not performed. Treatment included discontinuation of daptomycin and initiation of glucocorticoids, which resulted in rapid clinical recovery. Clinicians should suspect AEP when patients present with nonspecific respiratory symptoms while on daptomycin. Early diagnosis is crucial to prevent worsening symptoms, avoid unnecessary testing, and provide early discharge. The mainstay of treatments is discontinuation of daptomycin and glucocorticoid therapy.

## Introduction

Daptomycin is a lipopeptide antibiotic used primarily against Gram-positive organisms such as methicillin-resistant *Staphylococcus aureus* (MRSA) and vancomycin-resistant *Enterococcus* (VRE). Acute eosinophilic pneumonia (AEP) is a rare condition characterized by an excess of eosinophils in the interstitial and alveolar spaces, which is frequently linked to exposure to substances like inhalants, non-steroidal anti-inflammatory drugs, tobacco smoke, and, in rare cases, daptomycin. Daptomycin-induced AEP can be life-threatening if not recognized promptly. Its diagnosis can be challenging due to nonspecific signs and symptoms that overlap with more common infectious etiologies [1]. Associated risk factors generally include a longer duration of treatment and higher total cumulative doses [2,3]. It is, in fact, the leading cause of drug-induced eosinophilic pneumonia, with a prevalence ranging between 4.8% and 15% [4,5]. Bronchoscopy provides a definitive diagnosis, typically with an elevated eosinophil count exceeding 25% on bronchoalveolar lavage (BAL) [6]. We emphasize the need for an updated, well-organized diagnostic and therapeutic approach.

## Case presentation

An 80-year-old Caucasian male with a history of type II diabetes mellitus and coronary artery disease, who recently started on intravenous daptomycin (8 mg/kg daily) following amputation for osteomyelitis, presented three weeks later with acute dyspnea and mild fever that hindered his ability to perform daily tasks. On initial exam, he appeared in distress with a temperature of 100.6°F, blood pressure of 130/96 mmHg, heart rate of 95 beats/min, and respiratory rate of 22 breaths/min. He was found to be hypoxic with oxygen saturation of 83% on room air and was initiated on 7 L/min of supplemental oxygen via nasal cannula, resulting in an improvement to 95%. Chest examination was notable for diffuse expiratory rhonchi. The remainder of the examination was within normal limits. Initial investigations with complete blood count and metabolic panel were normal. Blood and respiratory cultures were collected. The respiratory PCR panel, as in Table 1, was unremarkable.

**Table 1:** Respiratory PCR panel results showing negative findings, effectively ruling out common bacterial, viral, and atypical infectious etiologies. MRSA: methicillin-resistant *Staphylococcus aureus; *PCR: polymerase chain reaction

Organism	Result
Adenovirus	Not Detected
Coronavirus HKU1	Not Detected
Coronavirus NL63	Not Detected
Coronavirus OC43	Not Detected
Human metapneumovirus	Not Detected
Bordetella pertussis	Not Detected
Bordetella parapertussis	Not Detected
Chlamydia (Chlamydophila) pneumoniae	Not Detected
Mycoplasma pneumoniae	Not Detected
Human rhinovirus/enterovirus	Not Detected
Influenza A	Not Detected
Influenza B	Not Detected
Parainfluenza virus 1	Not Detected
Parainfluenza virus 2	Not Detected
Parainfluenza virus 3	Not Detected
Parainfluenza virus 4	Not Detected
Respiratory syncytial virus	Not Detected
SARS-CoV-2	Not Detected
MRSA	Not Detected

**Figure 1:**
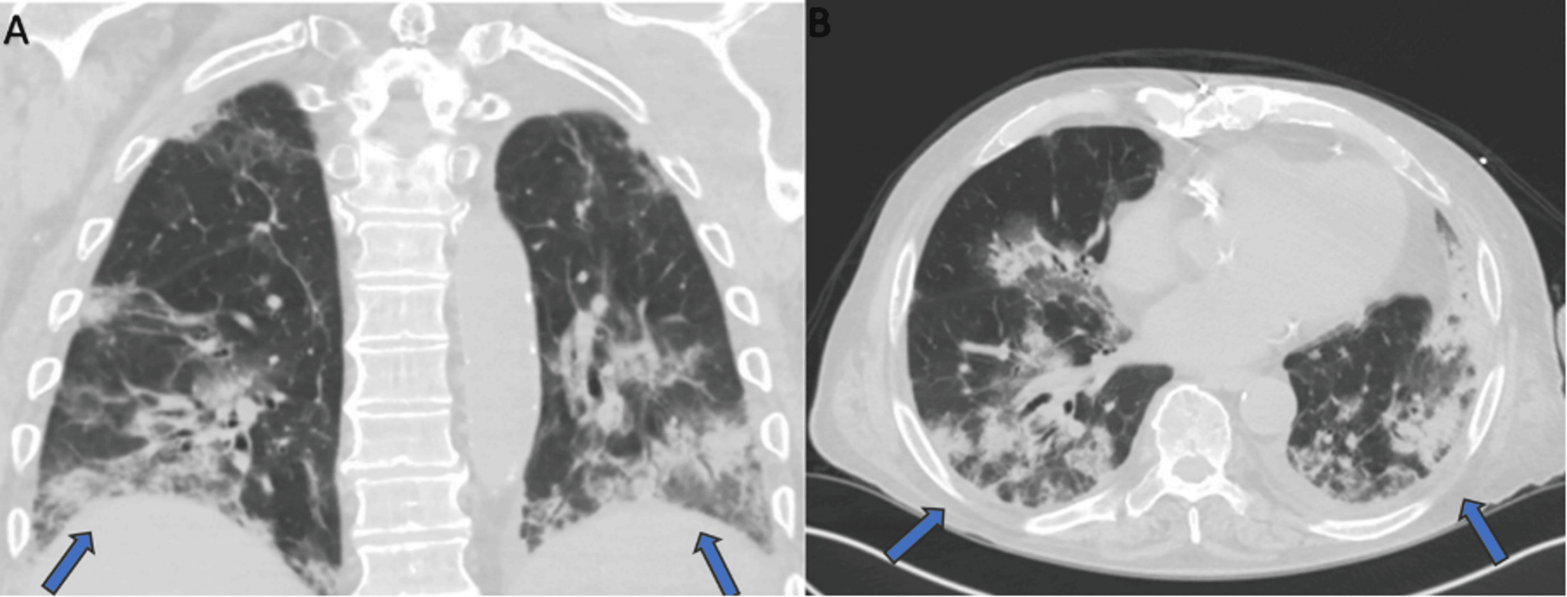
Chest CT showing patchy bilateral alveolar and ground-glass opacities (arrows), which may represent acute eosinophilic pneumonia. A: coronal view; B: axial view

In addition, *Legionella, Streptococcus pneumoniae, *histoplasma, and aspergillosis urine antigens were collected and were negative at day one of admission. Serum beta 1,3-D-glucan was also tested, with results that came back at 12 pg/ml (reference <60 pg/ml). A CT scan of the chest showed bilateral multifocal opacities, which were more noticeable towards the periphery (Figure 1).

Initial concern was for community-acquired pneumonia; hence, the patient was started on intravenous cefepime and vancomycin, and he was admitted to the progressive care unit as a case of multifocal pneumonia. After careful consideration of the daptomycin time of initiation and his symptoms, we expanded our differential diagnoses to include daptomycin-induced AEP. Blood and respiratory cultures remained negative for 48 hours. Consequently, antibiotics were de-escalated and stopped at day three of admission. Given our high clinical suspicion and after excluding other infectious etiologies, daptomycin was also discontinued, and the patient was administered 125 mg of intravenous methylprednisolone, resulting in a significant improvement in respiratory status within 24 hours. Bronchoscopy was deemed unnecessary, as the risk outweighed the benefit. So, BAL was not performed, and this is a limitation of our case. Eventually, the patient was diagnosed with a possible daptomycin-induced AEP and was discharged on ambient air with a prescription of 80 mg of prednisone daily for one week, with a gradual decrease of 10 mg per week thereafter. In addition, Bactrim double strength was prescribed three times per week for *Pneumocystis jirovecii* pneumonia (PJP) prophylaxis. On follow-up eight weeks after discharge, he had no recurrence of symptoms.

## Discussion

Several cases have described daptomycin-induced AEP. Although the pathophysiology remains incompletely understood, current evidence suggests that daptomycin interacts with pulmonary surfactant, potentially serving as an antigen that activates alveolar macrophages and initiates an inflammatory cascade leading to eosinophilic infiltration and lung injury [1]. Associated risk factors for developing this condition generally include longer duration of treatment and higher total cumulative doses of daptomycin [2,3]. According to the Food and Drug Administration (FDA), the diagnosis of daptomycin-induced AEP is defined as definite, probable, or unlikely based on specific criteria that include exposure to daptomycin, fever, dyspnea with increased oxygen demand or need for mechanical ventilation, new infiltrates on chest X-ray or CT scan, a BAL typically showing greater than 25% eosinophils, and clinical improvement following daptomycin discontinuation [4,5]. If all six criteria are met, the diagnosis is considered definitive. The full FDA diagnostic criteria are summarized in Table 2.

**Table 2:** FDA criteria for diagnosing daptomycin-induced acute eosinophilic pneumonia (AEP). A diagnosis is considered definitive when all six criteria are met, including BAL with >25% eosinophils. A diagnosis is considered probable when BAL shows <25% eosinophils or when peripheral eosinophilia is present. BAL: bronchoalveolar lavage

Definitive	Probable	Possible	Unlikely
Daptomycin exposure	Daptomycin exposure	Daptomycin exposure	Daptomycin exposure
Dyspnea with increased oxygen demand or need for mechanical ventilation	Dyspnea with increased oxygen demand or need for mechanical ventilation	New infiltrate on chest X-ray or CT scan	
New infiltrate on chest X-ray or CT scan	New infiltrate on chest X-ray or CT scan		
BAL with > 25% eosinophils	BAL with < 25% eosinophils or peripheral eosinophilia		
Improvement following daptomycin discontinuation	Improvement following daptomycin discontinuation		
Fever			

In our case, the patient fulfilled four of the FDA criteria, including exposure to daptomycin, dyspnea with increased oxygen demand, new infiltrates on imaging, and clinical improvement after discontinuation of therapy, but did not undergo BAL nor have documented peripheral eosinophilia. Therefore, the diagnosis aligns with the possible category of daptomycin-induced AEP [5,6]. Although definite diagnostic criteria typically include greater than 25% eosinophils on BAL, the impact of this procedure on outcomes is uncertain when there is high clinical suspicion. In this case, bronchoscopy was not performed as we thought the risk outweighed the benefit. In addition, the prompt clinical recovery following discontinuation of daptomycin along with glucocorticoid therapy supported our diagnosis. Therefore, diagnosis was solely based on the clinical context and imaging.

## Conclusions

Recognizing daptomycin-induced AEP requires careful consideration of the timing between the start of medication and the onset of symptoms. The discontinuation of daptomycin and the starting of glucocorticoids are the mainstays of the therapy. There is no consensus regarding the dose or length of glucocorticoid therapy, and this is yet to be studied. We emphasize the need for an updated, well-organized diagnostic and therapeutic approach.
